# Imaging diagnosis and staging of pancreatic ductal adenocarcinoma: a comprehensive review

**DOI:** 10.1186/s13244-020-00861-y

**Published:** 2020-04-25

**Authors:** Khaled Y. Elbanna, Hyun-Jung Jang, Tae Kyoung Kim

**Affiliations:** Joint Department of Medical Imaging, University Health Network, Mount Sinai Hospital and Women’s College Hospital, University of Toronto, Toronto, ON Canada

**Keywords:** Pancreatic cancer, Tumor resectability, Treatment response, Computed tomography, Magnetic resonance imaging

## Abstract

Pancreatic ductal adenocarcinoma (PDAC) has continued to have a poor prognosis for the last few decades in spite of recent advances in different imaging modalities mainly due to difficulty in early diagnosis and aggressive biological behavior. Early PDAC can be missed on CT due to similar attenuation relative to the normal pancreas, small size, or hidden location in the uncinate process. Tumor resectability and its contingency on the vascular invasion most commonly assessed with multi-phasic thin-slice CT is a continuously changing concept, particularly in the era of frequent neoadjuvant therapy. Coexistent celiac artery stenosis may affect the surgical plan in patients undergoing pancreaticoduodenectomy. In this review, we discuss the challenges related to the imaging of PDAC. These include radiological and clinical subtleties of the tumor, evolving imaging criteria for tumor resectability, preoperative diagnosis of accompanying celiac artery stenosis, and post-neoadjuvant therapy imaging. For each category, the key imaging features and potential pitfalls on cross-sectional imaging will be discussed. Also, we will describe the imaging discriminators of potential mimickers of PDAC.

## Key points

- Main pancreatic duct stricture is a red flag for small PDAC.
- Pancreatic and bile duct dilatation can be absent in uncinate process PDAC.
- Tumor-vessel relationship is a key parameter in the management of PDAC.
- Preoperative diagnosis of celiac artery stenosis is important in patients undergoing pancreaticoduodenectomy.
- Some key imaging features may help discriminate PDAC from its mimics.

## Background

Pancreatic ductal adenocarcinoma (PDAC) is associated with a poor prognosis with a dismal 5-year survival rate of 6–7%, most importantly due to delayed clinical presentation at an advanced stage when the tumor invades the surrounding structures or metastasizes [1, 2]. Approximately, 30% of patients with PDAC present with locally advanced cancer and the majority have metastasis either at the time of diagnosis or later during the disease course [3]. Currently, there are no reliable blood markers that can help an early detection of PDAC, and early-stage tumor is usually asymptomatic. Furthermore, PDAC can be missed in abdominal CT examinations performed for other reasons before clinical presentation. Subtle pancreatic abnormalities may be detected on the retrospective review of CT images in these patients [4, 5]. Improvements in imaging modalities and techniques have intensified radiologists’ role in the management of PDAC. Multi-detector CT with the availability of thinner slices, multi-planar reformat, and 3D images helps a detailed assessment of the tumor and tumor-vessel relationship [6]. Also, advanced MR imaging and endoscopic ultrasound (EUS) are often used as problem-solving tools in tumor detection and staging [4].

Small isoattenuating PDAC, which can only manifest as a main pancreatic duct (MPD) stricture, can be easily missed on CT [7]. Uncinate process PDAC is often clinically silent and can be overlooked on imaging particularly at its early stage due to absent biliary or pancreatic ductal dilatation [8]. The grey-zone of tumor resectability is often debated during multidisciplinary tumor boards as the criteria for borderline resectability are variable among different institutions. Therefore, the radiologists should be able to itemize the key findings according to their clinical impact [9]. It is challenging to interpret the imaging appearance of PDAC after neoadjuvant therapy due to difficulties in differentiating necrosis, fibro-inflammation or edema from the residual tumor on imaging [10].

In this pictorial review, we will discuss several challenges in imaging diagnosis of PDAC in terms of tumor detection, preoperative evaluation, and assessment of response to treatment on imaging. Also, we will describe the imaging features to differentiate PDAC from other benign and malignant conditions that appear similar on imaging.

## Discussion

### Small and isoattenuating PDAC

**Fig. 1:**
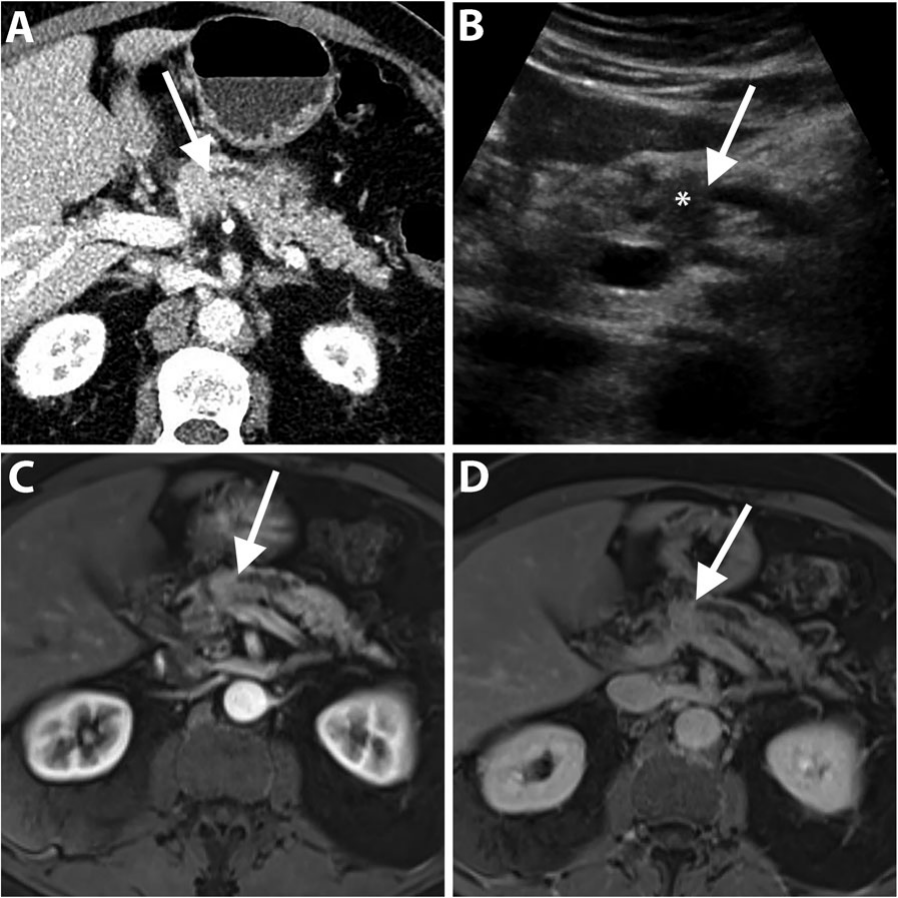
A 72-year-old man with isoattenuating PDAC. Axial pancreatic phase CT image (**a**) shows MPD stricture (arrow) at the pancreatic body without visible mass. Transverse transabdominal ultrasound image (**b**) shows a hypoechoic mass (asterisk) at the site of MPD stricture (arrow). Dynamic contrast-enhanced MR images using fat-suppressed T1-weighted sequence in the arterial (**c**) and portal venous (**d**) phases show MPD stricture (arrow) but fail to demonstrate a distinct mass. Subsequent pancreaticoduodenectomy revealed PDAC

Conventional transabdominal ultrasound may be helpful to visualize an isoattenuating pancreatic mass on CT [11]. On transabdominal US, PDAC is usually seen as an irregular hypoechoic mass associated with an abrupt cut-off of the main pancreatic duct (MPD) and upstream MPD dilatation [12] (Fig. 1). Transabdominal US has a sensitivity ranging from 75 to 89% for the detection of PDAC depending on operator experience, patient body habitus, and the effect of bowel gas on imaging quality [1]. In spite of these limitations, US is an easily accessible tool and frequently used in medical check-ups. In a multicenter study, US abnormalities were the clue to the diagnosis of early-stage PDAC in 91% and 41% of symptomatic and asymptomatic patients, respectively. The most commonly reported findings were MPD dilatation followed by pancreatic mass and MPD stricture [13].

CT is considered the initial imaging modality for evaluating patients with suspected PDAC. Pancreatic CT is usually performed with biphasic contrast-enhanced examination, including pancreatic phase typically at 40–50 s and portal venous phase at 65–70 s. PDAC usually has a dense fibroblastic stroma and, hence, typically appears as a hypoattenuating mass compared to normal pancreatic parenchyma during the pancreatic phase [7, 9, 14]. PDAC can be accurately diagnosed on CT with an overall sensitivity of 89% and specificity of 90% [15]. However, small and isoattenuating PDACs are challenging and can be overlooked on CT with a reported lower sensitivity of 58–77% for the detection of small (≤ 2 cm) tumors [16–18]. Isoattenuating tumors have a prevalence of 5.4–11% in all-size tumors and even higher (27%) in small (≤ 2 cm) tumors [7, 19, 20]. Noteworthy, isoattenuating tumors are more common among well-differentiated PDAC compared with poorly differentiated [7], and are associated with better survival rates after surgical resection. Isoattenuating PDAC tends to have lower tumor cellularity and less frequent tumor necrosis than hypoattenuating PDAC [19]. The evolving technology of Dual-energy CT technique may be helpful in the detection of subtle, small tumors by increasing the lesion conspicuity on low-keV virtual monoenergetic imaging reconstruction. This advantage is based on accentuating the attenuation difference between the hypovascular tumor and the surrounding parenchyma [21].

**Fig. 2:**
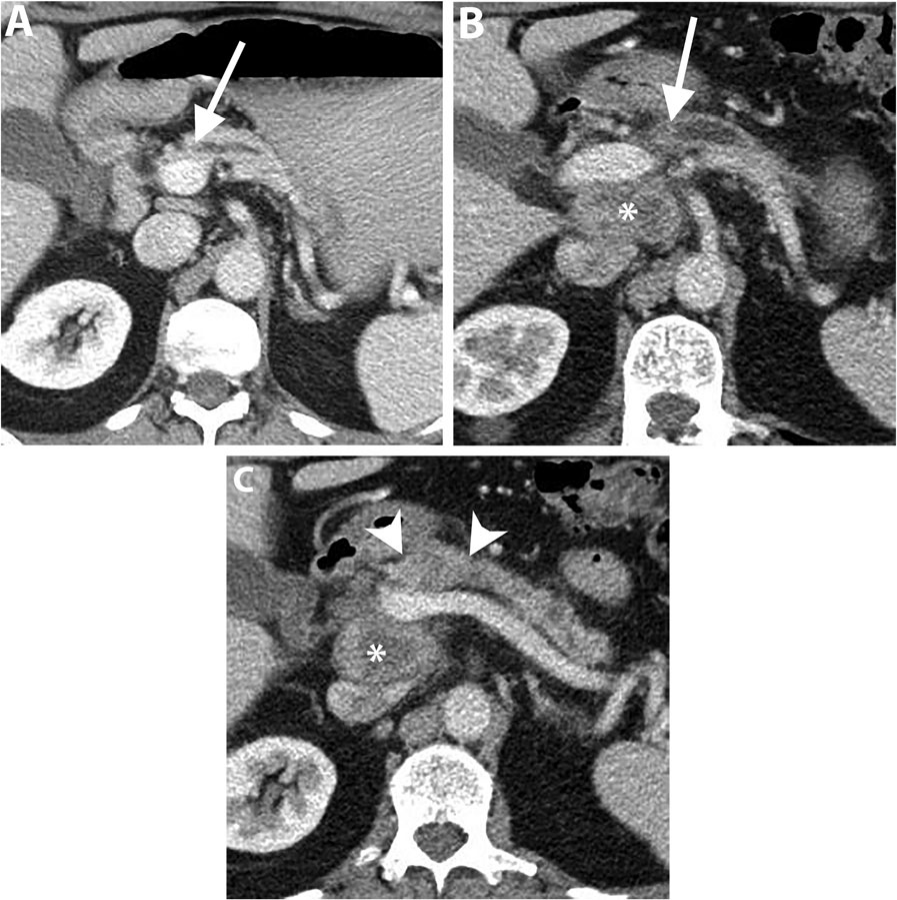
A 72-year-old man with PDAC. Axial portal venous phase CT image (**a**) shows MPD stricture (arrow) at the pancreatic body with upstream dilatation without visible obstructing mass. Axial portal venous phase CT images (**b** and **c**) obtained 6 months later show the progression of the MPD stricture (arrow) with worsened upstream ductal dilatation and pancreatic atrophy. There is a small hypoattenuating mass (arrowheads in **c**) associated with an enlarged necrotic metastatic portacaval lymph node (asterisk)

Early detection of subtle PDAC can be improved by identifying secondary signs that are seen in the majority (88%) of small isoattenuating tumors. The secondary signs include the following: (a) abrupt cut-off of MPD with or without upstream ductal dilatation; (b) distal pancreatic atrophy; (c) irregular pancreatic contour at the site of the tumor; (d) dilated MPD and CBD “double duct” sign; and (e) vascular encasement or narrowing [7, 22]. In a study by Gangi et al., a focal stricture and upstream pancreatic duct dilatation were the key CT findings in subtle PDAC. In retrospect, these findings were found in 50 and 7% of the patients 18 months and earlier, respectively, before the actual diagnosis of PDAC [23] (Fig. 2). Therefore, the secondary signs should be considered a red flag, requiring further evaluation with MR imaging or EUS rather than imaging follow up [7].

PDAC mostly appears hypointense to normal pancreas on fat-suppressed T1-weighted images and hypointense to isointense on post-contrast T1-weighted MR images [24]. The reported diagnostic accuracy of MR imaging has been shown to be equivalent to CT with a specificity of 89% [15]; however, it has an added value in detecting isoattenuating PDAC on CT [25]. A restricted diffusion is identified in PDAC on diffusion-weighted imaging (DWI) due to decreased extracellular space and increased cellularity and fibrosis within the tumor. DWI has a high diagnostic performance with reported sensitivity and specificity of 92–96% and 97–99%, respectively [26, 27]. Nevertheless, it has a limited diagnostic value in differentiating PDAC from mass-forming chronic pancreatitis due to an overlap in ADC values [28, 29].

EUS is of particular importance in patients with high clinical suspicion of PDAC without a detectable mass on CT, especially for small (≤ 2 cm) tumors. The reported sensitivity and specificity were 87% and 98%, respectively. PDAC appears as a hypoechoic lesion relative to the normal pancreatic tissue. In the absence of discrete mass, EUS has the advantage of obtaining a tissue biopsy targeted towards the area of focal pancreatic or common bile duct stricture [30].

The role of FDG-PET in early detection of PDAC remains controversial. High detection rates have been reported with a sensitivity of 81–100% for small (≤ 2 cm) PDAC [31, 32]. A large retrospective study, however, demonstrated a decline of the FDG-PET sensitivity to 50% for small tumors implying its low diagnostic yield in early-stage PDAC [33].

### Uncinate process PDAC

The uncinate process is a tongue-like extension from the inferior aspect of the pancreatic head that extends posteriorly behind the superior mesenteric vein (SMV) and artery (SMA). The incidence of uncinate process PDAC ranges from 2.5% to 10.7% of all PDAC [34]. Uncinate process is relatively distant from the pancreatic and common bile ducts, while it is closer to the SMA, SMV, and main portal vein (MPV) compared with the remaining pancreas. Owing to these particular anatomic features, uncinate process PDACs often have clinical manifestations and imaging characteristics dissimilar to other PDAC in the pancreatic head. Abdominal pain, rather than jaundice, is the most frequent presenting symptom in uncinate process PDAC, often leading to a late clinical presentation and diagnosis [8].

**Fig. 3:**
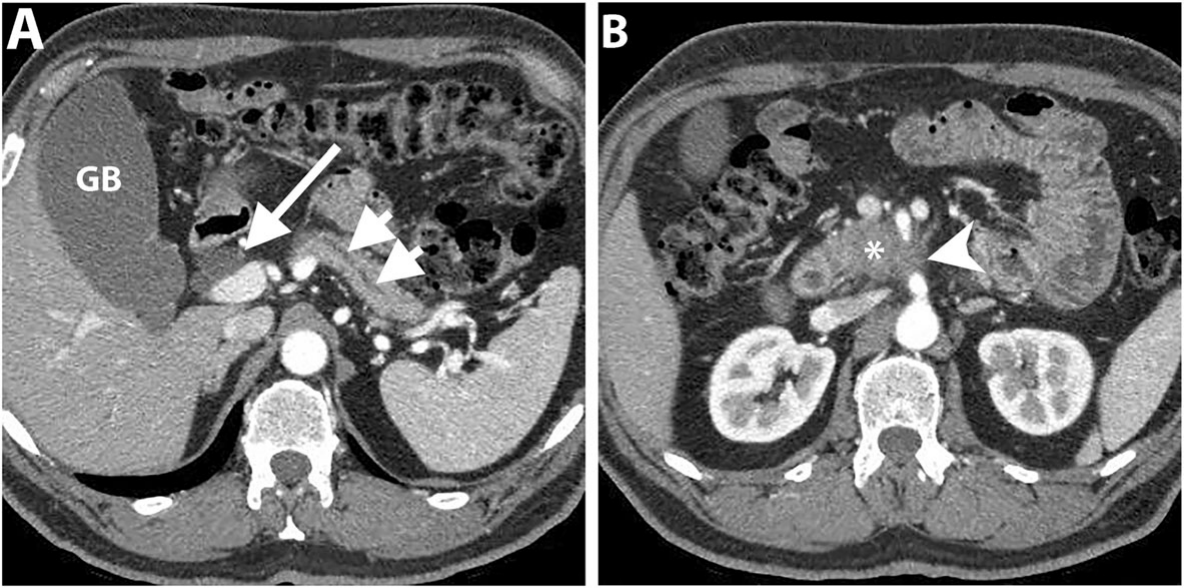
A 63-year-old man with locally advanced PDAC in uncinate process. Axial pancreatic phase CT images (**a** and **b**) show a mass (arrowhead) in the uncinate process encasing SMA with mild upstream dilatation of CBD (arrow) and MPD (short arrows) and associated with mildly distended gallbladder (GB)

**Fig. 4:**
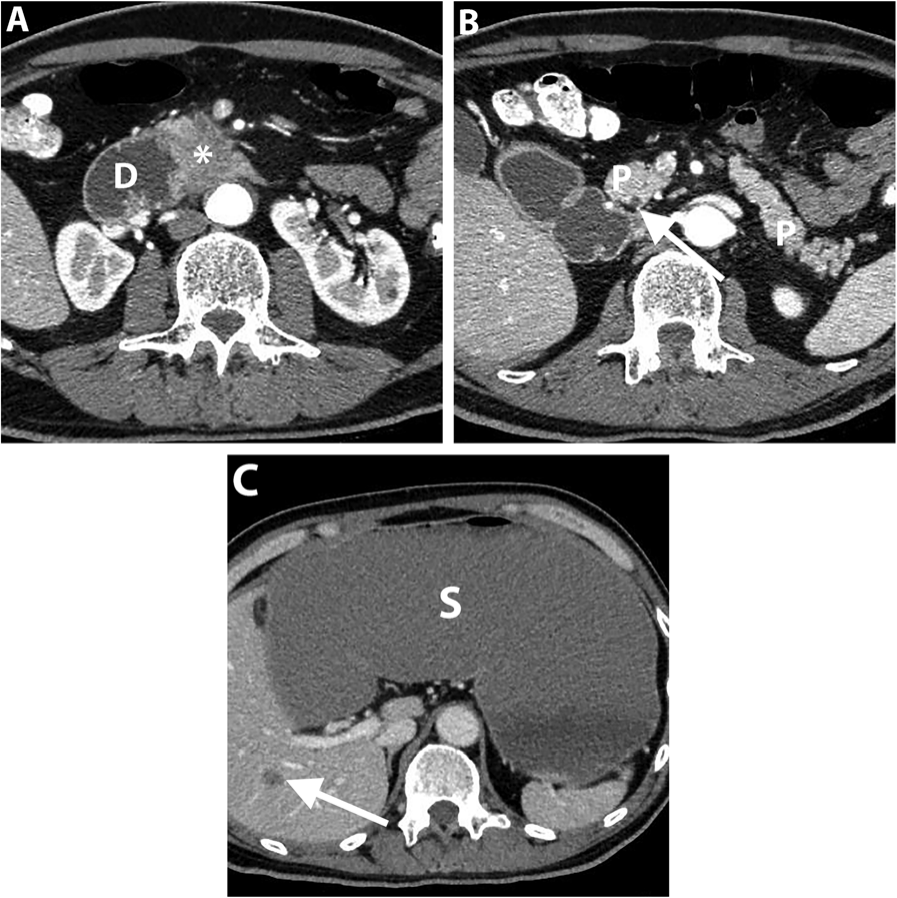
A 63-year-old man with uncinate process PDAC causing a duodenal obstruction. Axial pancreatic phase CT images (**a** and **b**) show a hypoattenuating mass (asterisk) arising from the uncinate process, infiltrating the third part of the duodenum (D) and abutting the anterior wall of the aorta. CBD (arrow) is normal in caliber. The pancreas (P) has no ductal dilatation or atrophy. Axial portal venous phase CT image (**c**) obtained 1 month later shows a marked distension of the stomach (S) due to duodenal obstruction and there is a new hypoattenuating metastatic liver lesion (arrow)

Uncinate process PDAC, compared to PDAC in other locations, is more frequently associated with vascular invasion, namely SMA, SMV, and MPV encasement (Fig. 3). Furthermore, a higher incidence of extrapancreatic perineural invasion has been reported [8, 35]. Duodenal invasion is also more common with a reported rate of 68% compared to 41% in other head PDAC [35]. As a result, a duodenal obstruction may develop in up to 9% of patients with uncinate process PDAC [8]. At CT and MRI, duodenal invasion is identified as a contiguous tumor extension within the duodenal wall and focal interruption of the normal mural enhancement of the duodenum [35, 36] (Fig. 4).

**Fig. 5:**
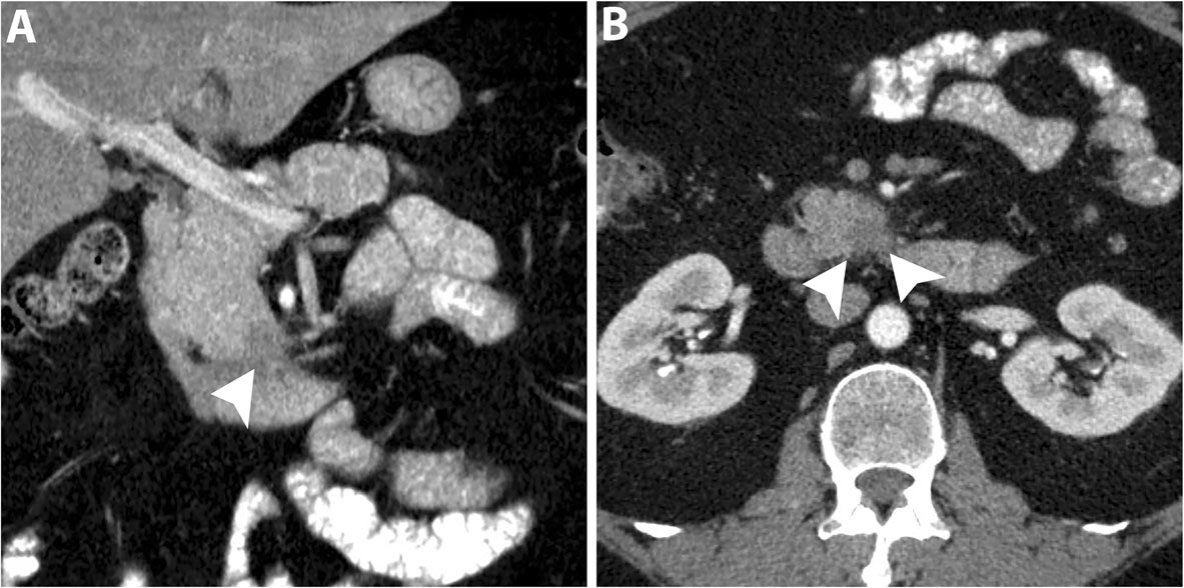
A 47-year-old man with uncinate process PDAC. Axial (**a**) and coronal (**b**) pancreatic phase CT images show a small hypoattenuating mass (arrowheads) in the uncinate process of the pancreas. Due to the location of the tumor, there is no CBD or MPD dilatation. EUS-guided biopsy revealed PDAC and the patient underwent pancreaticoduodenectomy

Early-stage uncinate process PDAC can be easily missed on imaging because of the subtlety of the tumor and absence of secondary signs of ductal dilatation (Fig. 5). Tamada et al. reported a 14% incidence of PDAC without secondary signs on preoperative CT of which 50% were located in the uncinate process [11].

### Evolving imaging criteria for tumor resectability

CT is the modality of choice for the assessment of vascular invasion with a specificity of 82–100% and sensitivity of 70–96% [37–40]. Biphasic pancreatic CT, performed with thin slice thickness (< 3 mm, preferably 0.5–1 mm if available) and multi-planar reformatting, is the optimal technique to evaluate the peripancreatic arteries and veins during the pancreatic and portal venous phases, respectively [9]. Axial, coronal, and sagittal reconstructions should be examined thoroughly to assess the tumor contact with the circumference and long axis of the vessels. Maximum intensity projections (MIP) images and volume-rendered images are useful in detecting subtle changes in vascular calibers [41]. MR imaging including MR angiography is an excellent alternative option with a sensitivity and specificity comparable to CT [1, 15, 39, 42]. EUS has a sensitivity of 72% and specificity 89% for the preoperative diagnosis of vascular invasion [43] with a higher sensitivity (94%) and specificity (89%) when using a contrast material [44]. However, EUS is not routinely recommended to assess vascular involvement due to variable hepatic arterial anatomy, high operator-dependence, and relative invasiveness [45].

Tumor abutment is defined as circumferential tumor contact ≤ 180° with the vessel, and tumor encasement refers to > 180° tumor contact with the vessel [9, 46, 47]. Teardrop sign, an altered shape of the affected vein on axial CT images due to tumor encasement or desmoplastic reaction, is highly associated with venous invasion [41, 48]. Morphological changes of the artery carry a higher risk of invasion compared with the vein. Tumor encasement of the arteries on CT has a sensitivity of up to 80% and a specificity of 98% for vascular invasion [41, 49].

Locally advanced tumors, in the absence of distant metastasis, are usually treated with chemoradiotherapy, whereas resectable tumors are usually treated with an upfront surgical resection, which is the only potentially curative treatment of PDAC [1, 2]. A borderline resectable (BR) tumor is an entity which fails to be classified under these two categories. This category is variable among different institutions particularly in defining the criteria related to venous invasion due to variations in vascular reconstruction surgeries [40]. In 2016, the International Association of Pancreatology (IAP) attempted to promote an international consensus to define borderline resectable PDAC (BR-PDAC) and included (1) anatomical, (2) biological, and (3) conditional criteria (Table 1) [50].

**Table 1:** The defining criteria of borderline resectability according to the International Association of Pancreatology (IAP) consensus (2016)

IAP consensus criteria for defining borderline resectability of PDAC (2016)	
Anatomical	• Higher likelihood of positive resection margin • Neoadjuvant therapy increases the probability for (R0) *Borderline venous* • > 180° tumor contact with SMV/PV or bilateral narrowing or occlusion without extension beyond the inferior border of the duodenum
	*Borderline arterial* • ≤ 180° tumor contact with SMA/CA without stenosis or deformity • Tumor abutment of CHA with no extension to proper hepatic artery and/or CA
Biological	• Suspicious but uncertain distant metastasis • Serum carbohydrate antigen (CA 19–9) > 500 U/ml
Conditional	• Performance status and comorbidities of the patients even considered even if the tumor is resectable

*SMV* superior mesenteric vein, *PV* portal vein, *CA* celiac artery, *CHA* common hepatic artery, *SMA* superior mesenteric artery

**Fig. 6:**
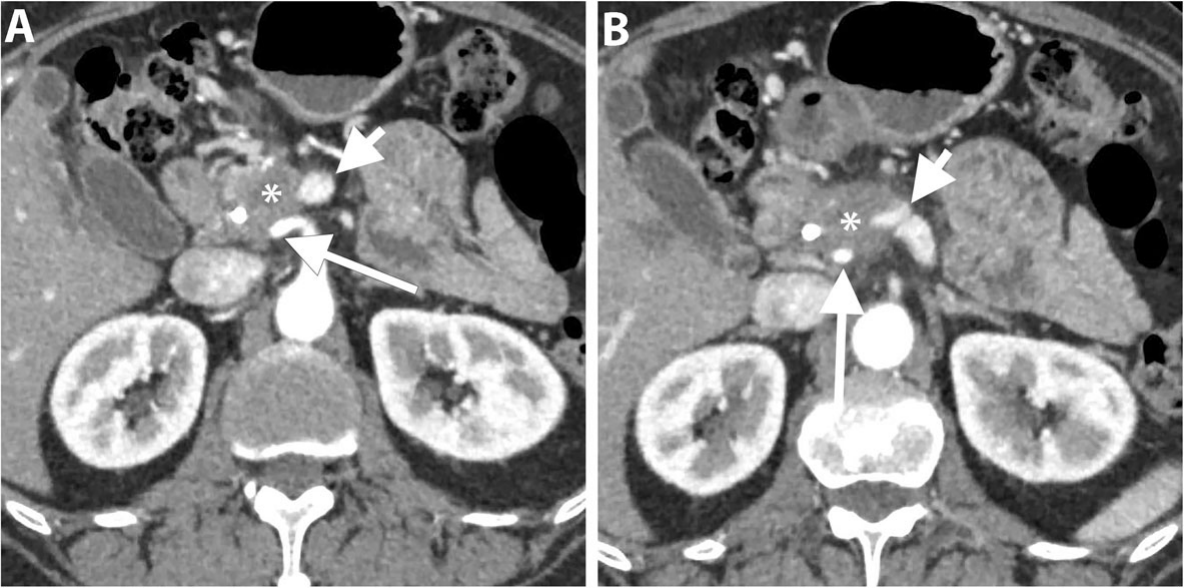
A 63-year-old man with PDAC. Axial pancreatic phase CT images (**a** and **b**) show a hypoattenuating mass (asterisk) in the pancreatic head with > 180° tumor contact with a replaced right hepatic artery (long arrow) and > 180° tumor contact with SMV (short arrow) with deformity of the vein lumen. The patient underwent a total pancreatectomy and vascular reconstruction after neoadjuvant therapy, but liver metastases developed one year after surgery

In IAP consensus 2016, slightly differing from National Comprehensive Cancer Network (NCCN) guidelines in 2019 (Table 2) [51], BR-PDAC is subdivided into venous-BR where the tumor only involves PV/SMV, and arterial-BR if the arteries are involved alone or together with the veins (Fig. 6). Furthermore, the absence of tumor contact with the first jejunal branch draining into SMV is excluded from the IAP criteria for BR-PDAC due to anatomic variations of the jejunal branches and difficult identification on CT. Instead, the inferior border of the duodenum has been considered as the anatomic landmark to assess the extent of venous invasion and to discriminate BR-PDAC from unresectable PDAC [50].

**Table 2:** The defining criteria of tumor resectability according to the National Comprehensive Network (NCCN) guidelines (2019) and the International Association of Pancreatology (IAP) consensus (2016)

Resectibility	NCCN (2019)	IPA consensus (2016)
Resectable	• No tumor-vessel contact	• Same
	• **≤** 180° tumor contact with SMV/PV WITHOUT venous contour irregularity	• Unilateral narrowing of the vein
Borderline resectable (veins)	• > 180° tumor contact with SMV/PV • ≤ 180° tumor contact with SMV/PV + venous contour irregularity or thrombosis if the vein is reconstructible • Tumor contact with IVC	• > 180° tumor contact with SMV/PV or bilateral narrowing or occlusion without extension beyond the inferior border of the duodenum
Borderline resectable (arteries)	• ≤ 180° tumor contact with CA/SMA	• ≤ 180° tumor contact with CA/SMA but without artery deformity or stenosis
	• Tumor contact with CHA WITHOUT extension to CA or HA bifurcation	• Same
	• Tumor contact with a variant arterial anatomy	• Not included
Unresectable	• Metastasis “including non-regional LN” • > 180° tumor contact with CA or SMA • Tumor contact with Aorta	• Same
	• Unreconstructible SMV/PV due to tumor invasion or bland/tumor thrombosis	• Occlusion or bilateral narrowing of SMV/PV extending beyond the inferior border of the duodenum
	• Tumor contact with the most proximal draining jejunal branch into SMV or the first jejunal SMA branch	• Not included

*SMV* superior mesenteric vein, *PV* portal vein, *CA* celiac artery, *CHA* common hepatic artery, *SMA* superior mesenteric artery

The presence of anatomic arterial variants increases the risk for intraoperative vascular injuries and postoperative complications such as hepatic ischemia, biliary anastomotic leak, and pseudoaneurysms. Hepatic arterial anatomic variations occur in 55–79% of the patients and include a replaced right or left hepatic artery, an accessory right or left hepatic artery, and a hepatomesenteric trunk, where the common hepatic trunk arises from the SMA [41, 48]. Preoperative diagnosis of these variations can aid in surgical planning and selecting the vascular reconstruction technique to reserve the aberrant artery and avoid vascular injury. The radiologist should report the arterial variant especially the presence of a replaced hepatic artery or hepatomesenteric trunk because they may determine tumor resectability. The report should also describe the absence or presence and degree of tumor contact with the aberrant artery [9, 41].

PDAC is deemed unresectable if there is a tumor encasement to CA/SMA, unfeasible reconstruction of SMV/PV, or tumor contact with the aorta, the most proximal jejunal SMV branch or the first jejunal SMA branch [51]. Due to its location, large PDAC has the potential to extend via multiple peritoneal and retroperitoneal anatomic planes and invade the adjacent structures including the stomach, spleen, colon, kidneys, and adrenals. The tumor invasion to these organs, if present, should be described in the radiological report [9, 52]. Multiplanar reformatting CT helps optimal chasing of the blood vessels as anatomic landmarks for tumor spread, for example, (a) the middle colic vessels for the transverse colon and mesocolon, (b) SMA and vein for the mesentery, (c) proper hepatic artery and PV for the hepatoduodenal ligament, (d) splenic vessels for splenorenal ligament, spleen and left kidney, and (e) left gastroepiploic vessels for the greater curvature of the stomach and splenic hilum [9, 52].

Metastatic disease from PDAC commonly affects the liver, peritoneum, lungs, and bones. Intraoperative detection of small liver or peritoneal metastasis is the most frequent cause (up to 55%) of aborted surgery in candidates with a preoperative CT diagnosis of a resectable tumor [53, 54]. Missed liver metastases are usually small and are found in the subcapsular area of the liver, raising the possibility of being a form of locoregional peritoneal dissemination rather than hematogenous metastasis [55]. MR imaging is more sensitive for the depiction of small liver metastasis with a sensitivity of 90─100% for MR imaging with either gadobenate dimeglumine or gadoxetic acid compared with 70─76% for CT [56]. Also, MR with DWI has a greater specificity for characterizing indeterminate liver lesions identified on CT; metastases usually demonstrate intermediate hyperintensity on T2-weighted MR images with restricted diffusion on DWI and rim enhancement on dynamic contrast-enhanced images [55, 57]. Early peritoneal metastases are often occult and too small to identify by the currently available imaging modalities. Therefore, unexplained peritoneal thickening or ascites should raise the suspicion of peritoneal carcinomatosis. In a suspected peritoneal disease, laparoscopic staging may be considered in patients with resectable PDAC [54, 56].

### Imaging findings after neoadjuvant therapy

Neoadjuvant chemoradiotherapy (CRT) has been used to improve the chance of tumor-free resection margin (R0) for borderline resectable PDAC or to downstage non-metastatic locally advanced tumors. CRT can result in downstaging in approximately 30% of patients with subsequent radiologic and/or histologic response [58]. CRT is increasingly used recently in multiple cancer institutions even for patients who have a resectable PDAC although the potential benefits and drawbacks are still to be determined [59].

**Fig. 7:**
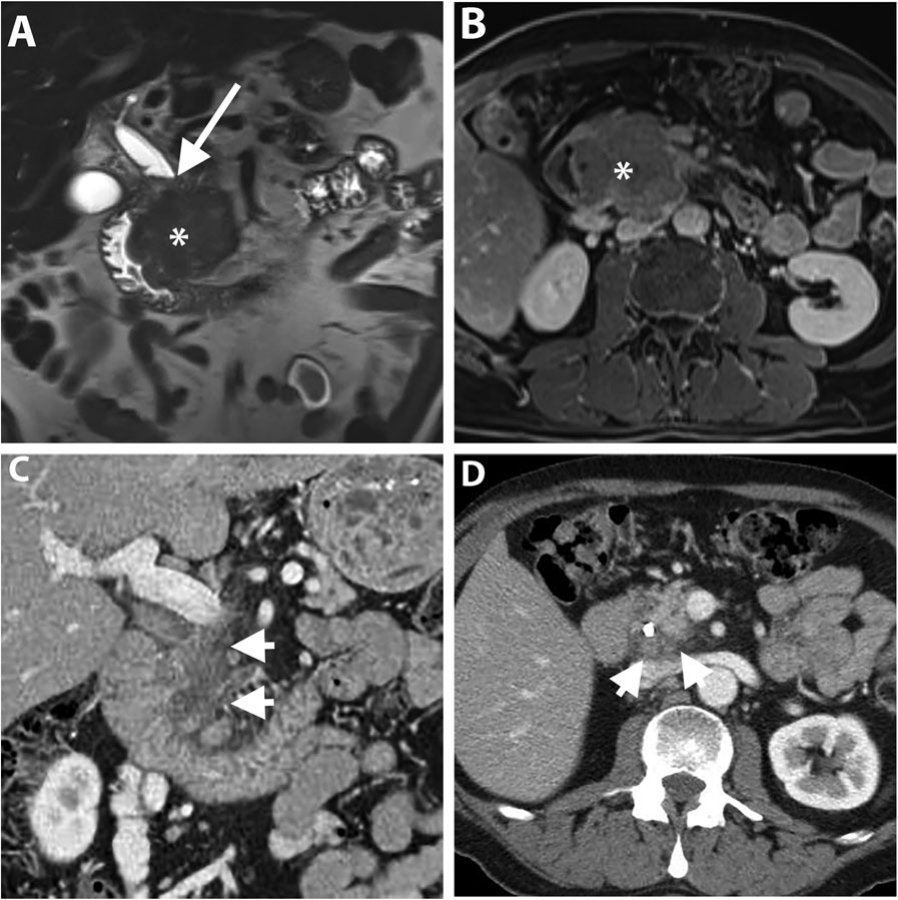
A 64-year-old man with PDAC of the head. Coronal T2-weighted MR image (**a**) shows a large low-intermdiate signal intensity pancreatic head mass (asterisk) causing upstream CBD dilatation (long arrow). Axial contrast-enhanced T1-weighted image in the portal venous phase (**b**) shows a hypoenhancing pancreatic head mass abutting SMV. Coronal (**c**) and axial (**d**) portal venous phase CT images obtained 2 months later after neoadjuvant therapy show a significant reduction of the tumor bulk (short arrows). The patient subsequently underwent pancreaticoduodenectomy, and pathology revealed extensive neoadjuvant treatment effect on PDAC with only 1 cm residual tumor and negative resection margin. No recurrence has been reported for 3 years

**Fig. 8:**
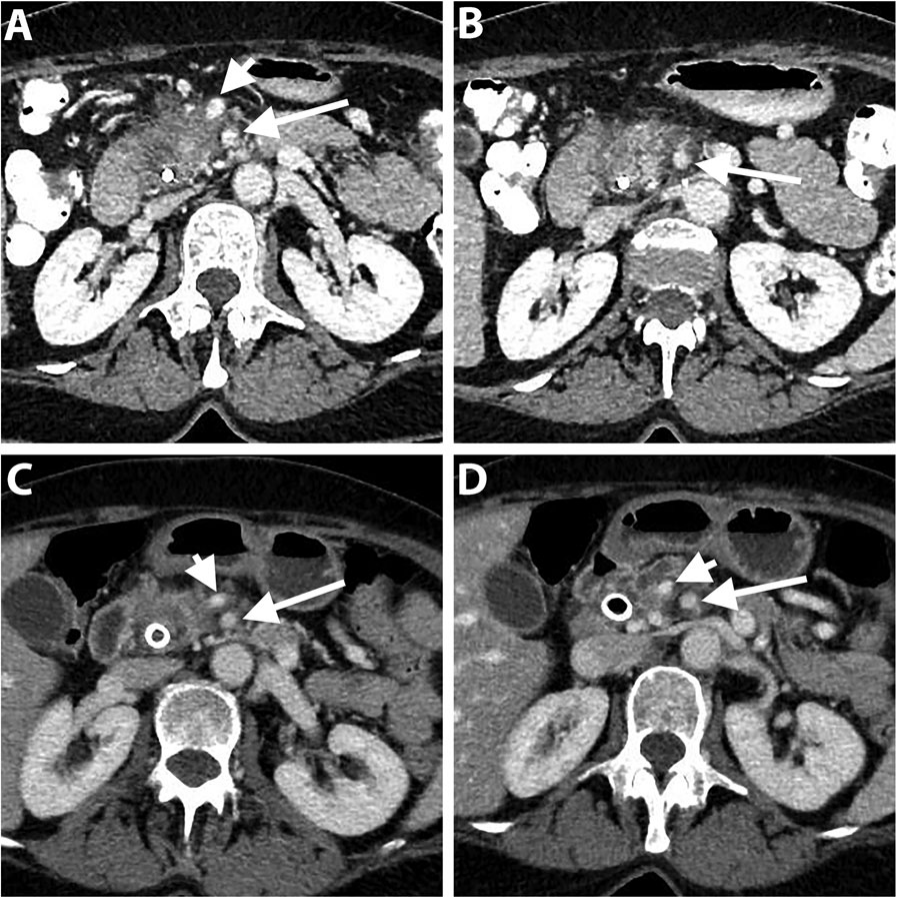
A 62-year-old woman with PDAC of the head. Axial portal venous phase CT images (**a** and **b**) show an ill-defined hypoattenuating mass encasing SMA (long arrow). SMV demonstrates a teardrop sign (short arrow) and is completely obliterated at a higher level (not shown). Axial portal venous phase CT images (**c** and **d**) obtained 2 months after neoadjuvant therapy show interval reduction of the tumor size and tumor contact with SMA (long arrow) and SMV (short arrow). The patient underwent pancreaticoduodenectomy and pathology revealed no residual invasion to SMA

It is challenging to assess response to CRT on CT [60, 61]. Morphological criteria including tumor size, attenuation, and contact with the vessels have been proposed to assess response to CRT. However, tumor size can be overestimated on CT due to treatment-related changes such as necrosis and edema and the change in tumor size has no significant correlation with tumor-free resection margin (R0). Similarly, the change in tumor attenuation is of limited value to predict resectability due to the inability to differentiate necrosis, fibro-inflammation or edema from residual tumor tissue. Therefore, alterations in tumor size and attenuation on CT have low accuracy to monitor tumor response to treatment [10, 61]. However, in a study by Cassinotto et al., a reduction of tumor-vessel contact was significantly associated with R0 resection regardless of the tumor size reduction or the extent of tumor-vessel contact. A partial reduction of the tumor contact with SMV/PV, SMA, CA, or hepatic artery was associated with R0 resection in 91% of patients, suggesting that this finding might be considered an indication for surgical resection in suitable candidates [10]. Therefore, the change of the degree of tumor contact with the circumference of the peripancreatic vessel may be particularly important (Figs. 7 and 8).

There is an emerging role of DWI in tumor restaging after neoadjuvant therapy in different abdominal malignancies such as rectal and cervical cancers [62, 63]. PDAC demonstrates high signal intensity on high b-value DWI and lower signal intensity on the apparent diffusion coefficient (ADC) map as compared to the normal parenchyma [64]. In a recent study, DWI quantitative parameters were evaluated in PDAC patients receiving chemotherapy and provided an early and accurate discrimination between responders and non-responders. A progressive reduction in DW-volume was observed in the responders’ group [65]. Dalah et al. analyzed the changes in ADC values following the initiation of CRT in patients with resectable and borderline resectable PDAC. The authors found that ADC values were significantly higher in post-CRT as compared to pre-CRT with a significant correlation with the tumor pathologic response [66]. Therefore, DWI may serve as a useful imaging biomarker to predict tumor response and select PDAC patients who could benefit from neoadjuvant therapy [64]. However, there is still a limited evidence about the value of DWI. Also, the decreased image quality of DWI and lack of reproducibility of ADC values may cause restrictions on its clinical utility [67].

FDG-PET-CT can be a useful tool to predict the outcomes in patients receiving CRT. Results have shown that the greater the difference between the pre- and posttreatment maximum standard uptake values (SUVmax), the better survival rates and the longer progression-free survival [68].

### Celiac artery stenosis

Celiac artery (CA) stenosis is found in 2.0–7.6% of patients undergoing pancreaticoduodenectomy [69]. It can be due to external compression by the median arcuate ligament (MAL) or intrinsic stenosis by atherosclerotic disease. The clinical management differs according to the underlying cause of stenosis [70, 71]. The assessment of CA stenosis is particularly important in patients who are candidate for pancreaticoduodenectomy because postprocedural termination of the collateral flow from the SMA to CA branches may put the patient at risk of hepatic arterial ischemia. CA evaluation on sagittal reformatted images should be included in the checklist in all preoperative CT for pancreaticoduodenectomy [71, 72].

CT angiography is helpful to detect CA stenosis, identify the underlying etiology, and map the collateral pathways [73, 74]. In MAL compression syndrome, CT angiography demonstrates a superior notch of the CA, located around 5 mm from its origin with a characteristic “J,” “U,” or hook-shaped appearance. The superior notch of CA may also be seen in normal individuals and, therefore, performing CT angiography during expiration helps to avoid false-positive results. Additional CT features include post-stenotic dilatation and enlarged peripancreatic collateral arteries. Atherosclerotic stenosis, in contrast, typically affects the ostium and is associated with intimal calcifications of CA [74, 75].

**Fig. 9:**
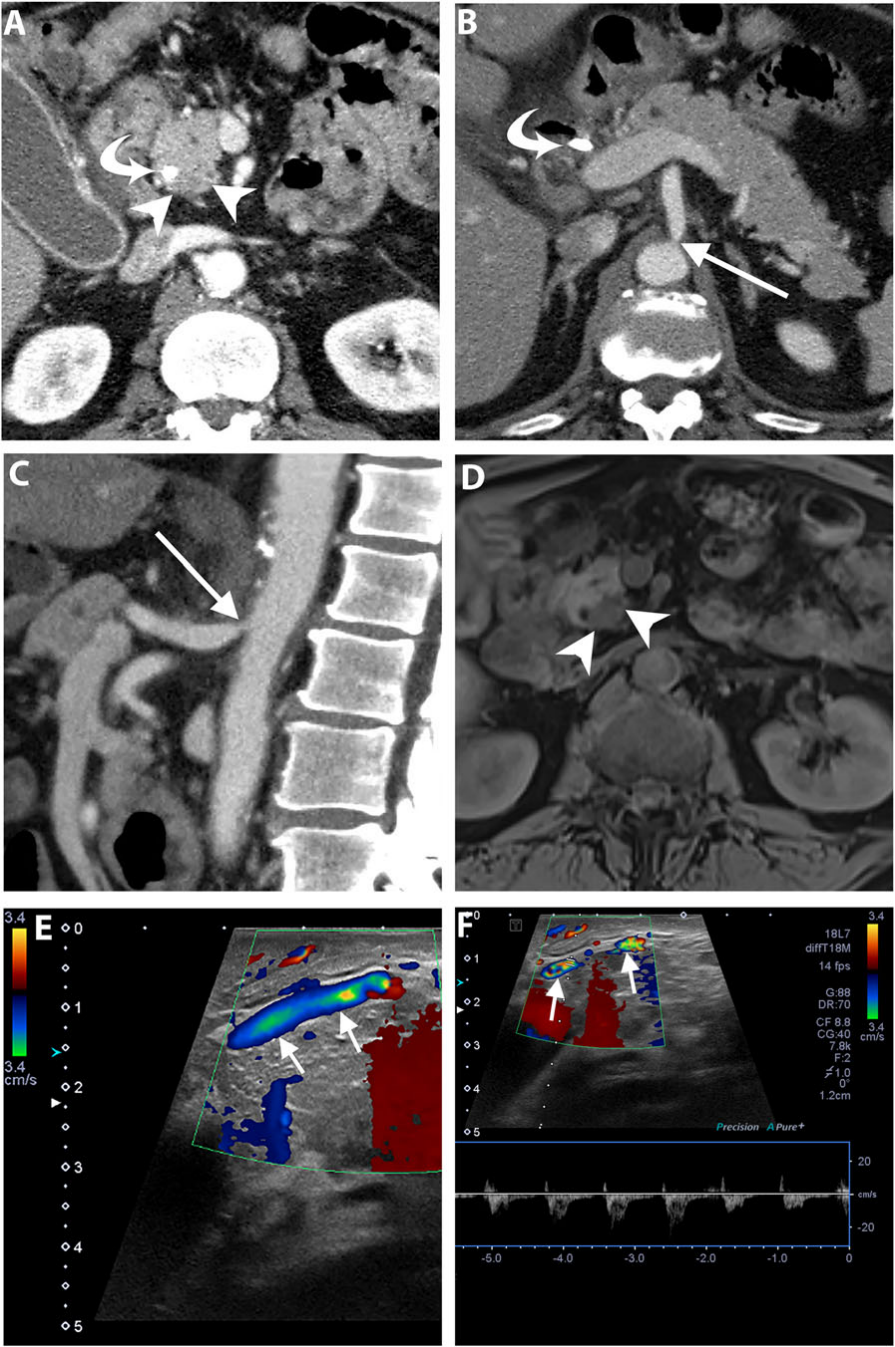
A 60-year-old man undergoing preoperative imaging for PDAC. Axial (**a** and **b**), and sagittal (**c**) pancreatic phase CT images show ostial stenosis of the celiac artery (arrow) due to atherosclerotic disease. The patient has a biliary stent (curved arrow) and PDAC is identified as a subtle hypoattenuating lesion (arrowheads). Axial fat-suppressed T1-weighted MR image (**d**) clearly demonstrates PDAC as a hypointense mass. Intraoperative Doppler ultrasound (**e** and **f**) shows a significant celiac artery stenosis by demonstrating caudocranial/reversed blood flow in the gastroduodenal artery, denoting its significant contribution to the hepatic arterial supply. The patient subsequently underwent pancreaticoduodenectomy after celiac artery stenting

In CA stenosis, the major collateral pathways between the CA and SMA include pancreaticoduodenal arcades and dorsal pancreatic arteries that are seen in 95% and in 76%, respectively. The anterior pancreaticoduodenal arcade runs along the pancreaticoduodenal groove anteriorly as a continuation of the gastroduodenal artery (GDA), while the posterior one courses posterior to the distal common bile duct. The dorsal pancreatic artery arises from the splenic, celiac or common hepatic artery, and courses posteromedial to the SMV [74]. CT angiography is useful to assess the degree of stenosis but unable to reflect its hemodynamic significance. Hence, intraoperative Doppler US can be used to assess hepatic arterial flow after clamping of the GDA. A preserved Doppler signal indicates an effective collateral flow, whereas a significant reduction in the hepatic arterial flow justifies for arterial reconstruction [71, 72, 76] (Fig. 9).

### Mimics of PDAC

Variable conditions may appear similar to PDAC on imaging including benign abnormalities such as autoimmune and groove pancreatitis, and focal fat infiltration and malignant lesions such as neuroendocrine tumor, lymphoma, and metastasis.

**Fig. 10:**
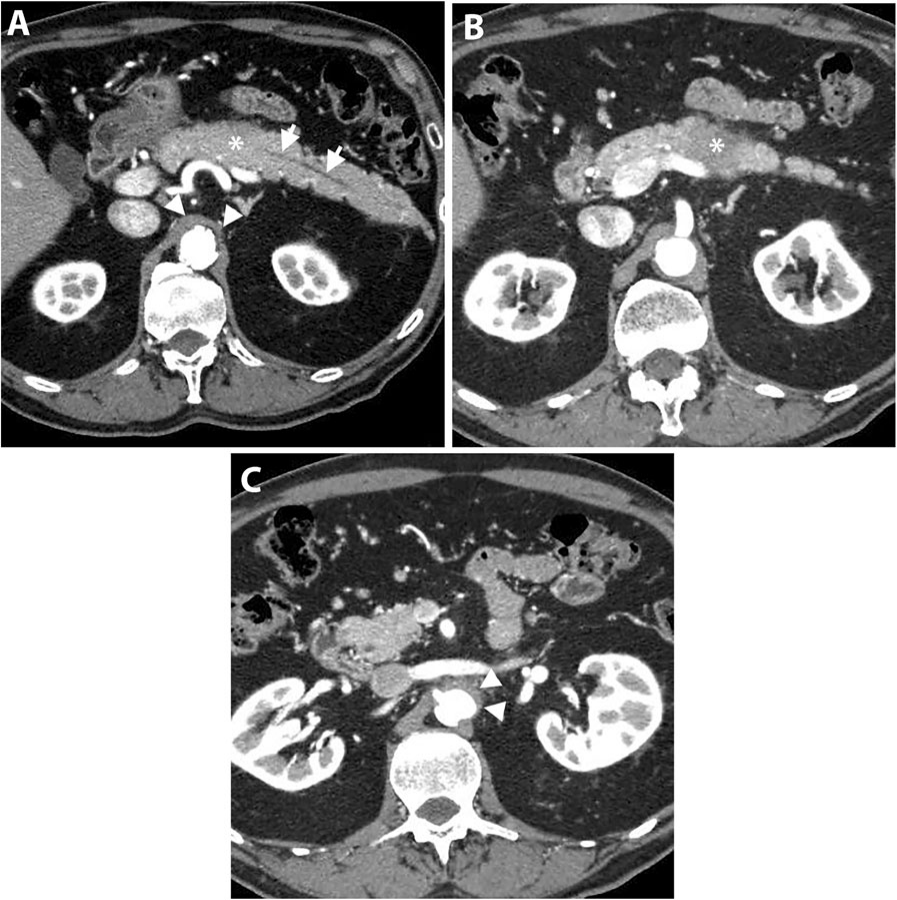
A 71-year-old man with a mass-forming AIP. Axial (**a**–**c**) pancreatic phase CT images show two ill-defined, mass-like lesions (asterisk) in the pancreatic body without significant MPD dilatation (arrows). There is associated rind of periaortic soft tissue thickening (arrowheads) representing IgG4-related retroperitoneal fibrosis

Autoimmune pancreatitis (AIP) is a great mimicker of PDAC and accounts for 2–3% of surgical resections for clinically suspected cancers; therefore, differentiating both entities is critical [77]. In diffuse AIP, the pancreas is diffusely enlarged “sausage shape” with loss of pancreatic lobulation and a capsule-like rim “halo” of hypoattenuation on CT or hypointensity on MR imaging [78]. Focal “mass-forming” AIP is a less common type and appears, similar to PDAC, as an irregular ill-defined mass-like abnormality; therefore, it is quite challenging to differentiate both entities. However, the presence of relatively long stricture, visible duct within a mass, multifocal strictures, and absence of substantial upstream pancreatic duct are more observed in focal AIP rather than PDAC [78–80] (Fig. 10). Furthermore, the retention of contrast during the delayed phase of postcontrast MR imaging is more frequent and distinct in AIP [80–83]. This pattern of enhancement is thought to be due to preserved acinar cells with mild fibrosis, whereas PDAC is completely replacing the normal pancreatic tissue by tumor cells with abundant fibrous stroma [83]. Collateral evidence of extrapancreatic IgG4-related disease is another important clue for diagnosing AIP. Several organs can be affected such as secondary sclerosing cholangitis with biliary stricture, bilateral renal mass-like lesions, retroperitoneal fibrosis, and sclerosing mesenteritis [78].

**Fig. 11:**
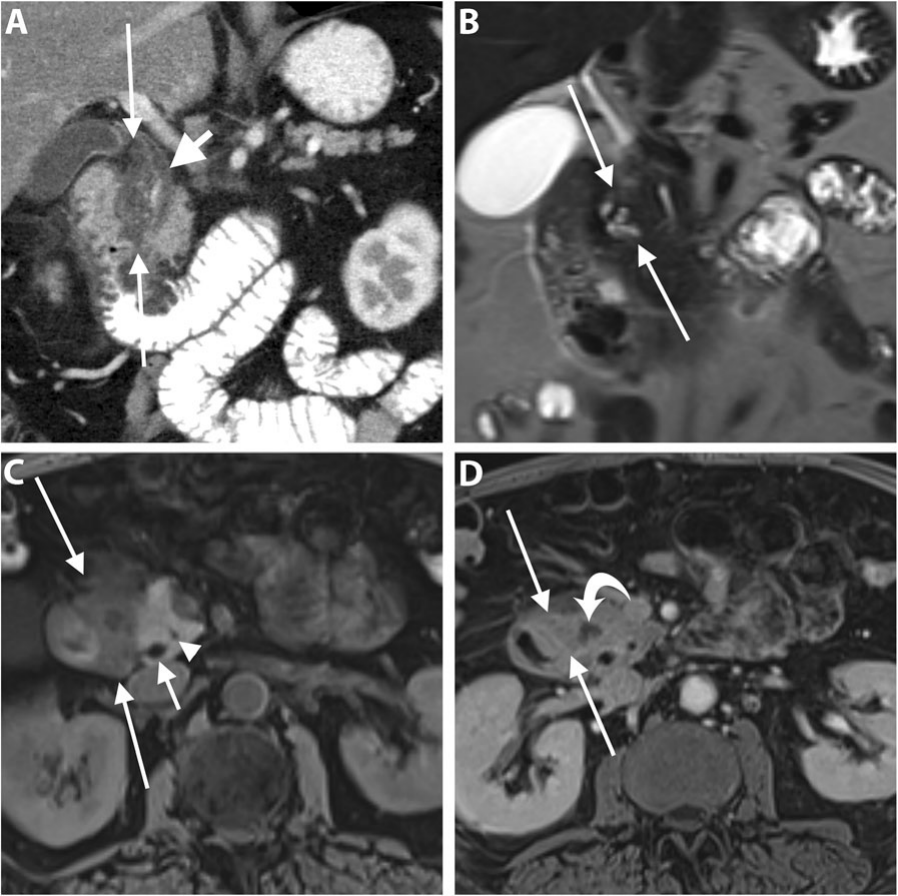
A 60-year-old woman with a segmental form of groove pancreatitis. Coronal portal venous phase CT image (**a**) shows a hypoattenuating sheet-like area in the pancreaticoduodenal groove (between long arrows) associated with mural thickening and luminal narrowing of the descending duodenum. CBD (short arrow) is displaced medially by the inflammatory process and tapers distally. MR images with coronal T2 HASTE sequence (**b**), axial fat-suppressed T1 sequence (**c**), and axial contrast-enhanced fat-suppressed T1 sequence of the delayed phase (**d**) show the pancreaticoduodenal groove abnormality (between long arrows) containing multiple tiny cysts along the duodenal wall with high T2-signal intensity and a sheet of fibro-inflammatory tissue with low T1-signal intensity, and delayed enhancement. Non-enhancing tiny pseuodocyst is noted (curved arrow). CBD (short arrow) and MPD (arrowhead) are not dilated. The patient has improved on subsequent follow-up

Groove pancreatitis (GP) is an uncommon specific entity of chronic pancreatitis affecting the groove between the pancreatic head, duodenum, and common bile duct, commonly affecting young men and associated with alcohol abuse [84]. The inflammation can be limited to the groove in the pure form of GP or extends to the pancreatic head in the segmental form [85]. PDAC and GV are quite difficult to distinguish due to similar features including low signal intensity on fat-suppressed T1-weighted images, intermediate to high signal intensity on T2-weighted MR images, and hypovascularity during the early phase of contrast-enhanced CT and MR imaging with variable degrees of delayed enhancement during the delayed phase [85, 86]. The key imaging features are mainly depicted on MR imaging and include (a) cystic changes around an accessory pancreatic duct in association with hyperenhancing, thickened wall of the descending duodenum; (b) smooth long stricture of the intrapancreatic CBD without marked upstream biliary dilatation; and (c) displaced CBD and GDA away from the duodenal lumen due to pancreaticoduodenal groove inflammatory tissue [84, 86–88] (Fig. 11). Definitive distinction of GP from PDAC may require EUS-guided tissue biopsy or fine-needle aspiration cytology. Although, the fibrotic tissue is present in both conditions, adding to the diagnostic uncertainty [84].

**Fig. 12:**
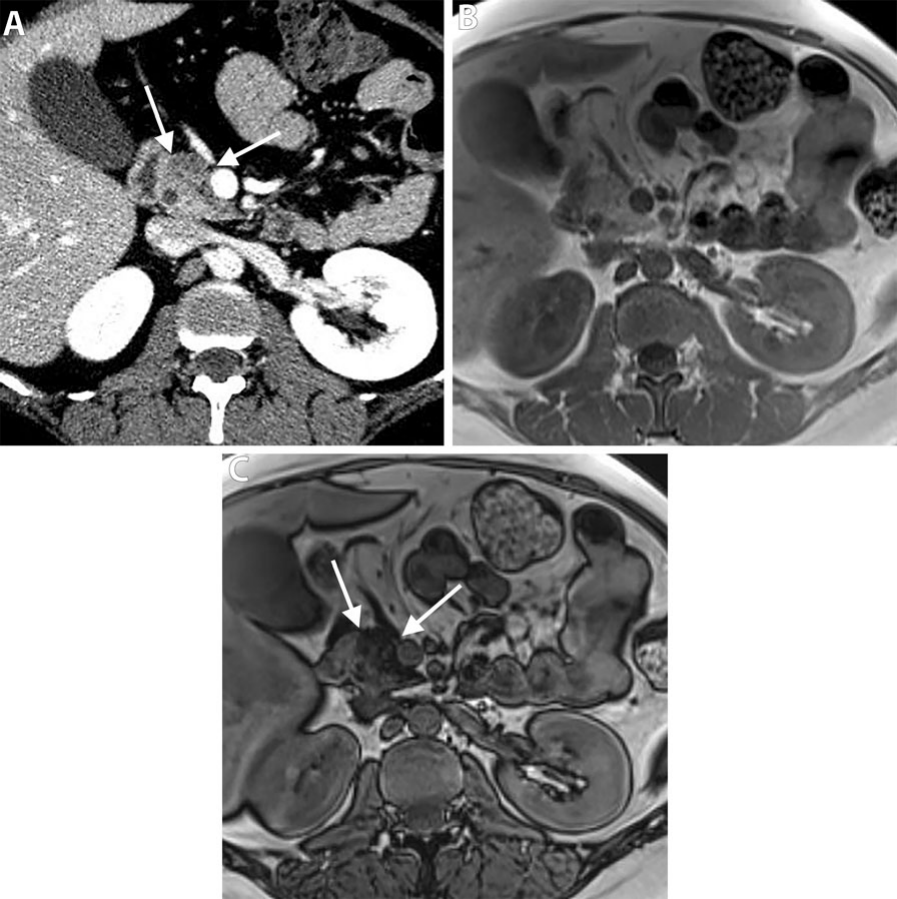
A 39-year-old woman with focal fat infiltration of the pancreatic head. Axial portal venous phase CT image (**a**) show low-attenuation area (arrows) in the pancreatic head with a tongue-like extension just posterior to SMV. No mass effect or MPD dilatation. Note the normal attenuation parenchyma around the CBD. Axial chemical shift MR images show no abnormality at in-phase sequence (**b**) and drop of signal of the same area (arrows) at opposed phase sequence (**c**) consistent of microscopic fat in focal fat infiltration

Focal fat infiltration of the pancreatic parenchyma reflects an uneven deposition of adipose tissue and often involves the anterior part of the pancreatic head with sparing of its posterior part and the area around the common bile duct [89]. On ultrasound, the focal fat sparing area is differentiated from hypoechoic mass by preserved course and caliber of CBD and sharp demarcation with the anterior hyperechoic zone of fat infiltration [90]. Pancreatic focal fat infiltration can be differentiated from PDAC at CT by the presence of a distinct border between the affected anterior portion and normal pancreatic tissue around the common bile duct, and absence of pancreatic ductal obstruction [91, 92]. MR imaging demonstrates a drop of signal intensity in the out-of-phase sequence, differentiating it from PDAC [91, 93] (Fig. 12).

**Fig. 13:**
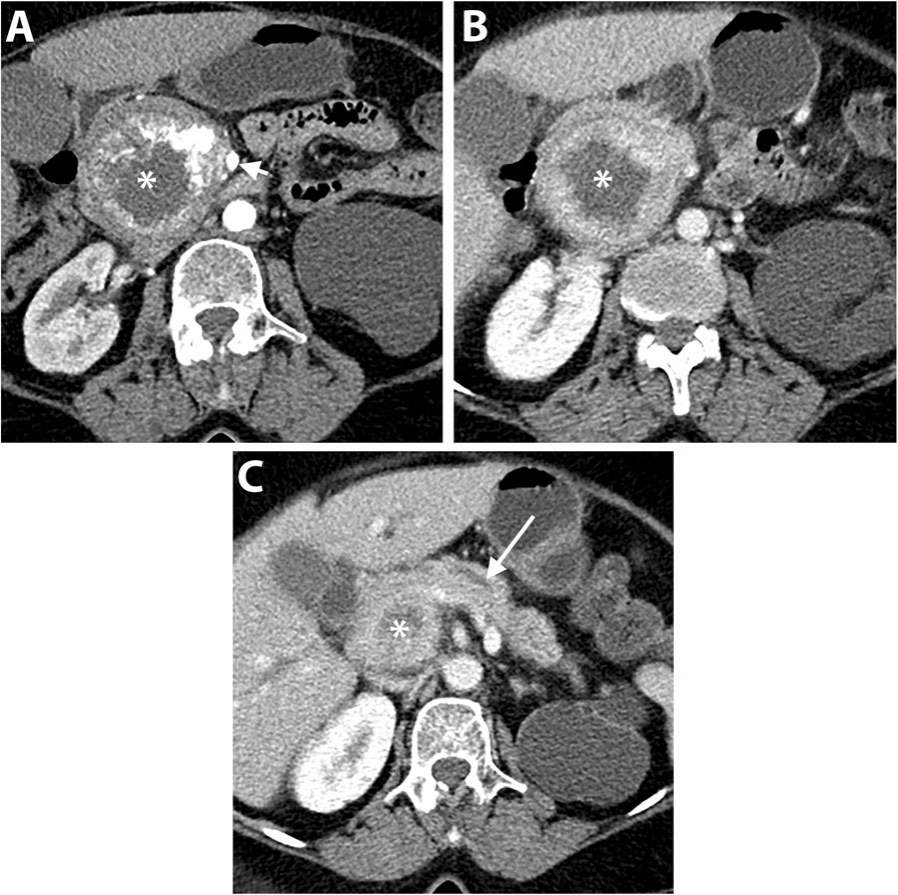
A 53-year-old man with a pancreatic neuroendocrine tumor of the head. Axial arterial phase (**a**) and portal venous phase (**b**) and (**c**) CT images show a well-defined mass (asterisk) with peripheral hypervascularity and central cystic area. Only mild dilatation of the MPD (arrow) due to external compression by the tumor, rather than ductal origin of the tumor. Histopathology revealed pancreatic endocrine tumor

Pancreatic neurendocrine tumors (PNETs) account for 1–3% of all pancreatic neoplasms, most commonly in the fourth–sixth decades of life [94, 95]. In contrast to PDAC, PNETs tend to show a well-circumscribed mass, iso-to hyperenhancing relative to the normal pancreas and less frequently associated with upstream MPD dilatation and distal pancreatic atrophy [94, 96, 97] (Fig. 13).

**Fig. 14:**
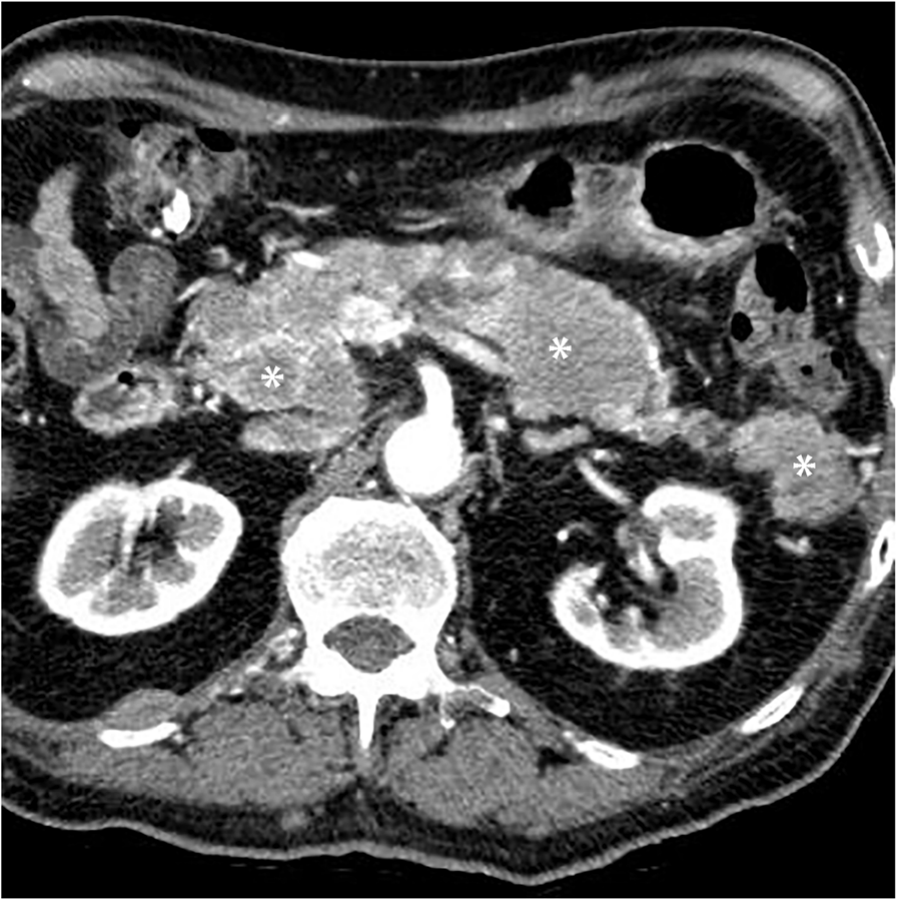
A 72-year-old man with papillary thyroid cancer metastasis involving the pancreas. Axial pancreatic phase CT image shows multiple enhancing masses (asterisks) involving the entire pancreas

Metastases to the pancreas is relatively uncommon accounting for 2–5% of malignant lesions, and the majority are from renal cell carcinoma followed by breast, lung, colorectal, and melanoma. Metastatic disease can present as solitary mass in 50–75%, diffuse infiltrative mass in 15–44% or multiple masses in 5–10 % [98] (Fig. 14). Metastases from renal cell and hepatocellular carcinomas are typically hypervascular and readily differentiated from the hypovascular PDAC; however, metastatic masses may attain larger size and develop central necrosis [99]. On the contrary, hypovascular metastases from the lung, breast, and colorectal cancers are quite similar to PDAC. Ductal involvement is not a reliable discriminatory criterion as it can also occur in metastasis and cause upstream pancreatic duct dilatation and distal pancreatic atrophy. Peripancreatic vascular invasion is rarely seen in metastatic disease [100]. A known history of primary malignancy, co-existing extrapancreatic metastasis, and multiplicity of pancreatic lesions can help in the diagnosis of metastasis. Otherwise, biopsy may be required if the lesion remains indeterminate [99].

Pancreatic lymphoma is rare accounting for less 0.5% of all pancreatic tumors and 2% of extranodal lymphoma. It can be primary in origin or, more commonly, secondary to extension from the peripancreatic lymph nodes. Non-Hodgkin lymphoma is the most frequent of pancreatic lymphoma. Morphologically, lymphoma can present as a focal mass, arising from the pancreatic head in 80% of cases; or a diffuse pancreatic enlargement simulating pancreatitis [99]. Lymphoma can be quite similar to PDAC on CT and MR imaging [101, 102]. Nonetheless, significant MPD dilatation is absent in lymphoma even with a sizable tumor. Moreover, lymphoma tends to encase the nearby vessels without significant invasion or occlusion and more frequently associated with infrarenal retroperitoneal lymphadenopathy [99, 102].

## Conclusion

Missed imaging diagnosis of PDAC can be minimized by increasing awareness of the secondary signs identified in subtle or isoattenuating tumors, prompting further diagnostic workup rather than follow-up imaging. Uncinate process PDAC can be easily missed at its early stage due to the lack of pancreatic and bile duct dilatation. By using different imaging modalities the radiologists can play a pivotal role in determining tumor resectability, aiding proper surgical planning and evaluating tumor response to treatment. It is also important for the radiologist to know the mimics of PDAC to avoid unnecessary surgery for benign entities such as focal fat infiltration, autoimmune, and groove pancreatitis, and to arrange for proper treatments in malignant tumors such as PNET, lymphoma, and metastasis.
